# Application of droplet digital PCR for quantitative detection of *Spiroplasma citri* in comparison with real time PCR

**DOI:** 10.1371/journal.pone.0184751

**Published:** 2017-09-14

**Authors:** Yogita Maheshwari, Vijayanandraj Selvaraj, Subhas Hajeri, Raymond Yokomi

**Affiliations:** 1 USDA-ARS, San Joaquin Valley Agricultural Sciences Center, Parlier, CA, United States of America; 2 Citrus Pest Detection Program, Central California Tristeza Eradication Agency, Tulare, CA, United States of America; University of Helsinki, FINLAND

## Abstract

Droplet digital polymerase chain reaction (ddPCR) is a method for performing digital PCR that is based on water-oil emulsion droplet technology. It is a unique approach to measure the absolute copy number of nucleic acid targets without the need of external standards. This study evaluated the applicability of ddPCR as a quantitative detection tool for the *Spiroplasma citri*, causal agent of citrus stubborn disease (CSD) in citrus. Two sets of primers, SP1, based on the spiral in housekeeping gene, and a multicopy prophage gene, SpV1 ORF1, were used to evaluate ddPCR in comparison with real time (quantitative) PCR (qPCR) for *S*. *citri* detection in citrus tissues. Standard curve analyses on tenfold dilution series showed that both ddPCR and qPCR exhibited good linearity and efficiency. However, ddPCR had a tenfold greater sensitivity than qPCR and accurately quantified up to one copy of spiralin gene. Receiver operating characteristic analysis indicated that the ddPCR methodology was more robust for diagnosis of CSD and the area under the curve was significantly broader compared to qPCR. Field samples were used to validate ddPCR efficacy and demonstrated that it was equal or better than qPCR to detect *S*. *citri* infection in fruit columella due to a higher pathogen titer. The ddPCR assay detected both the *S*. *citri* spiralin and the SpV1 ORF1 targets quantitatively with high precision and accuracy compared to qPCR assay. The ddPCR was highly reproducible and repeatable for both the targets and showed higher resilience to PCR inhibitors in citrus tissue extract for the quantification of *S*. *citri* compare to qPCR.

## Introduction

Spiroplasmas are motile, helical bacteria belonging to the Class Mollicutes, a group of micro-organisms having no cell wall and phylogenetically related to Gram-positive bacteria [1]. Among the many spiroplasma species, three are pathogenic to plants: *Spiroplasma citri*, *S*. *kunkelii*, and *S*. *phoeniceum*. The first-cultured and most-studied spiroplasma is *S*. *citri*, the causal agent of citrus stubborn disease (CSD) [2] and horseradish brittle root disease [3], and is transmitted in a circulative–propagative manner by several species of the leafhoppers such as *Circulifer tenellus* in California [4, 5] and *Circulifer haematoceps* in the Mediterranean area [6]. Natural Spread of CSD is limited by several factors such as: i) specificity of leafhopper species capable of supporting the persistent mode of vector transmission and ii) *Circulifer* leafhoppers prefer cruciferous plants and weeds and do not colonize citrus for long-term feeding or reproduction. As such, spatial and temporal patterns show CSD is a simple interest disease without tree-to tree spread. Proximity to row crop hosts of *S*. *citri* such as carrots can exacerbate spread in adjacent border rows of the citrus orchard [7, 8]. Symptoms of CSD include stunted growth, unseasonable growth flushes and blossoms, low yield, and small lopsided fruit. Symptom intensity can vary with citrus variety and age. All citrus cultivars are susceptible but sweet orange and grapefruit varieties are the most economically affected [9, 10, 11]. The CSD effect the production and fruit quality of sweet oranges and Navel oranges in commercial orchard in California [11] and growth, yield, fruit quality of frost Washington navel and Valencia oranges in Cyprus [12].

Diagnosis of CSD is challenging due to long incubation period of months to years from infection to development of disease symptoms, sporadic distribution and seasonal fluctuations of *S*. *citri* titer [13]. Isolation of *S*. *citri* is technically demanding and time consuming. Detection of *S*. *citri* now mainly relies on polymerase chain reaction (PCR) technologies involving the use of primers developed from sequences of housekeeping genes such as spiralin, 16s ribosomal RNA and adhesion genes [13,14,7]. An essential point for *S*. *citri* detection for epidemiology studies is a robust assay to provide unambiguous positive or negative results without the need for culturing except in cases when compulsory confirmation is required (e.g. regulatory). For this purpose, ddPCR was tested and compared to qPCR since it does not require a standard curve and is capable to detect a single copy of the target for which culturing would be unreliable.

Real time quantitative (q) PCR has been a standard molecular method for detection of *S*. *citri* and improved diagnosis was demonstrated by targeting the high copy number Prophage gene (SpV1-ORF1) [8]. The digital PCR (dPCR) concept has many potential advantages over qPCR, such as absolute quantification without dependence on external standard curves [15] and may be less affected by inhibitors. The dPCR has gained increasing popularity due to its so-called “droplet digital PCR” (ddPCR) system that has been termed a “third generation PCR”. In ddPCR, a mixture of target template and reaction mixtures are partitioned into thousands of micro droplets (theoretically up to 20,000 droplets). The ddPCR unit performs PCR on each reaction droplet, and the number of positive reactions, together with Poisson’s distribution, produces a direct, high confidence measurement of the original target concentration [16]. The absolute number of target nucleic acid molecules contained in the original sample before partitioning can be calculated directly from the ratio of positive to total partitions, using binomial Poisson statistics [17].

In this study, a single copy housekeeping gene (spiralin) and multi-copy Prophage gene (ORF1) were used to compare the linearity, dynamic range, sensitivity, tolerance to residual matrix inhibitors and diagnostic performance of qPCR and ddPCR assays. Wang et al. 2015 showed the improved detection of *S*. *citri* using ORF1 gene compare to other Spiroplasma genes (Spiralin, 16S rRNA, P58, SpV1-ORF3). The repeatability and reproducibility for ddPCR assays were done with *S*. *citri* infected field samples. The ddPCR proved to be a powerful new tool with higher accuracy and precision for detection of *S*. *citri* at low titer and, theoretically, at an early stage of infection. Unambiguous early detection of invasive pathogens like *S*. *citri* allows implementation of appropriate early control strategies to mitigate pathogen spread.

## Material and methods

### Pathogen isolation and cultivation

Petiole leaf midribs or fruit columella collected from Spring Navel/Carrizo from field plot near Ducor, California were excised, surface sterilized, and diced with a sterile razor blade in 5 ml of LD8 broth medium [18], passed through a 0.45-μm filter and incubated at 30°C. Presence of *S*. *citri* was confirmed after 3 to 14 days by examining 10 μl of culture medium under dark-field microscopy at ×400 to 1,000 for the presence of motile, helical spiroplasmas.

### Primers and probes

Primers and probes used in qPCR and ddPCR assays are shown in Table 1. The SP1 and ORF1 TaqMan probes were synthesized by labeling the 5′ -terminal nucleotide with 6-carboxy-fluorescein (FAM) reporter dye and the 3′ -terminal nucleotide with Minor groove binder/ nonfluorescent quencher (ThermoFisher Scientific, USA).

**Table 1:** Primer and probe sequences used for the qPCR and ddPCR assay for detection of *Spiroplasma citri*.

Target Gene (accession no.)	Primer/Probe name (location)	Sequence (5'-3')	Amplicon length	Reference
Spiralin (U13998)	SP1 F (209–232)	AAGCAGTGCAAGGAGTTGTAAAAA	79 bp (209–288)	[7]
	SP1 R (261–288)	TGATGTACCTTTGTTGTCTTGATAAACA		
	SP1 P (242–259)	6FAM/CAGCTGATTTTCAATTTG/MGB/NFQ		this study
SpV1-ORF1 Prophage (X51344)	ORF1F (777–798)	TGGCAGTTTTGTTTAGTCATCC	190 bp (777–966)	[8]
	ORF1R (946–966)	GGGTCTAAACGCCGTTAAAGT		
	ORF1P (922–941)	6FAM/TTGGGTTTGGTTATTCCATT/ MGB/NFQ		this study

### Standard curve

The spiralin gene (674 bp) and the prophage gene (533 bp) were amplified from *S*. *citri* DNA using Spiralin F/R and Prophage F/R primers, respectively (S1 Table). The amplicons were ligated in pGEM-T Easy vector (Promega) and transformed in JM-109 (Promega) separately. The plasmids isolated from white colonies were linearized using SpeI restriction enzyme (New England Biolabs, UK) and the concentrations were measured using dsDNA high sensitivity assay kit in Qubit 3.0 fluorometer (Thermofisher). Ten-fold serial dilutions were made using linearized SP1 and ORF1 plasmids to generate the standard curves used to assess analytical sensitivity, linearity and dynamic range of ddPCR and qPCR assays.

*S*. *citri* was cultured in 25 ml of LD8 broth to a log stage and adjusted 20,000 cells/ml and DNA was extracted from an 1 ml culture by cetyltrimethylammonium bromide (CTAB) [19] and used for 10-fold serial dilution ranging from 1ng to 10 fg per reaction were made in healthy citrus extract. Three replicates of each concentration were tested simultaneously in the same run. The linear relationship was produced by plotting the log DNA concentration against the quantitation cycle (Cq) (or cycle threshold).

### Quantitative real time PCR (qPCR)

The qPCR assay was performed in the CFX96 Real-Time System (Bio-Rad) in a final volume of 20 μl reaction that contained 10 μl of SsoAdvanced™ Universal Probes Supermix (Bio-Rad), 900 nM forward and reverse primers and 250 nM probe, 2 μl of *S*. *citri* DNA and final volume made up with double distilled water. The thermal cycling conditions consisted of initial denaturation at 95°C for 5 min, then 40 cycles of denaturation at 95°C for 10 s, and annealing at 57°C for 30 s. We have performed the qPCR with primer/probe concentration of 300nm/150nm and 900nm/250nm and obtained no significant Cq value changes. We used the same concentration of primer/probe for ddPCR as well, to maintain the same reaction conditions. Each run included *S*. *citri* positive and negative controls (DNA from healthy Navel sweet orange leaves and columella) along with a no template control (NTC). A standard curve was constructed for serial dilutions of *S*. *citri* plasmid DNA and included in all runs to produce quantitative results.

### Thermal gradient optimization of ddPCR assay

To determine the optimal annealing temperature for SP1 and ORF1, the thermal gradient ranging from 48, 49, 51, 53.9, 57.2, 60.1, 62.1, and 63°C was performed in the S100 Thermal cycler using the same amount of *S*. *citri* DNA and primers/probes concentrations (900nM/250nM).

### Droplet digital PCR assay (ddPCR)

The ddPCR reaction mixture (20 μl) contained 2x ddPCR Supermix for probes (no dUTP) (Bio-Rad), 900 nM of each forward and reverse primers, 250 nM of probe, and 2 μl of *S*. *citri* DNA. The reaction mixture was transferred in individual wells of disposable eight channel DG8 cartridge that was already preloaded in DG8 cartridge holder and the bottom wells were filled with 70 μl of droplet generation oil. The prepared cartridge was then placed into the QX 200 droplet generator. The prepared droplet emulsions were further loaded in a semi-skirted, PCR-clean 96 well plate (Eppendorf) using a multichannel pipette (Rainin, USA), by aspirating 40 μl from the DG8 cartridge. The plate was then heat sealed with pierceable foil using a PX1 PCR plate sealer (Bio-Rad) and PCR amplification was carried out in a S1000 thermal cycler (Bio-Rad). The thermal cycling for SP1 primers consisted of initial denaturation at 95°C for 10 min followed by 40 cycles of 94°C for 30 s (denaturation) and 54°C for 1 minute (annealing/elongation) with a ramp of 2°C/s and a final 10 min incubation at 98°C for enzyme deactivation. The same conditions were used for ORF1 primers except annealing temperature was 57°C. After thermal cycling, the plate containing the droplets was placed in a QX 200 droplet reader (Bio-Rad, CA, USA) for analysis.

### Field sample collection

Leaves and fruit samples were collected from four different quadrant branches from each of 50 trees near Ducor, Tulare County, California in November and tested in ddPCR and qPCR with SP1 and ORF1 primers. Because *S*. *citri* is a warm weather pathogen, sampling in this time period was not optimum. However, this plot had been sampled and tested numerous time previously for *S*. *citri* infection [7, 8]; therefore, each tree’s infection status was known. Two hundred mg of fresh fruit columella tissue from three fruits and ten leaf petioles per tree were excised and homogenized separately in 5 ml CTAB buffer [19] using a Homex 6 homogenizer (BioReba. AG, Reinach, Switzerland). The nucleic acid quality and quantity was measured using Qubit 3.0 (Thermofisher).

### Evaluation ddPCR and qPCR inhibition for residual matrices

Inhibition is often a problem when quantifying targets in field samples by qPCR-based methods. The influence of inhibitors with *S*. *citri* DNA during sample preparation was estimated for both ddPCR and qPCR assays by comparing their ability to quantify a constant amount of DNA in the presence of different quantity (1 μl to 5 μl) of citrus leaf petiole and fruit columella extract. The reactions were spiked with the same amount of *S*. *citri* plasmid DNA (SP1 and ORF1) and the influence of leaf and fruit extract were assessed relative to the mean measured signals in each sample with no added inhibitors (no-inhibition control, as double-distilled water plus *S*. *citri* plasmid DNA). To obtain citrus leaf petiole extracts, 0.5 g healthy citrus leaves were excised and homogenized in 10 ml TE buffer (pH 7.4) using a Homex 6 homogenizer (BioReba. AG) and centrifuged at 10,000x g for 10 min at 4°C. The fruit columella was excised and extract was collected as described above. The supernatants were collected to test the tolerance of both the qPCR and ddPCR assays to the citrus leaf petiole and fruit columella extracts.

### Reproducibility and repeatability of ddPCR with field samples

To assess the reproducibility (inter-assay variation) and repeatability (intra-assay variation) of ddPCR assays, triplicate experiments were performed with field collected *S*. *citri* infected fruit and leaf samples using SP1 and ORF1 primers and probes. The reproducibility was determined by measuring the samples in triplicate within the same experiment (to assess the intra-assay variation) and between three different assays (inter-assay variation). Coefficient of variation (CV) was calculated by standard deviation/mean.

### Data analysis

The standard curves and Cq values for qPCR were generated by Bio-Rad CFX Manager Software version 3.1. Linear regression of the qPCR standard curves was recalculated with Microsoft Excel software (Microsoft, USA). The Cq values were regressed against the logarithmically transformed copy number and DNA concentration. The qPCR amplification efficiency was estimated from the slopes of the standard curves using the equation E = 10^−1/slope^– 1. The ddPCR data were analyzed with QuantaSoft analysis software version 1.7 (Bio-Rad). The positive droplets containing amplified products were discriminated from negative droplets by applying a threshold above the negative droplets. The reactions with more than 10,000 accepted droplets per well were used for analysis. The copy number concentration of each sample was reported automatically by ddPCR software. The linear regression and P-value of the ddPCR assay was determined by plotting the measured copies of ddPCR and comparing them with expected values of serial dilution of plasmid DNA and bacterial cell culture DNA in Excel. The Poisson error and total error were calculated by QuantaSoft software. Poisson error was calculated for merged well, with contributions from subsampling and partitioning and total errors was calculated for replicates. Total error bars always greater than or equal to Poisson error bars for true instrument technical replicates. Receiver operating characteristic (ROC) curves were constructed to evaluate the diagnostic performance of the ddPCR and qPCR assays. True positive trees infected with *S*. *citri* were taken in to analysis for ROC curve based on the previous year’s data. Pearson Correlation and regression between the ddPCR and qPCR measurements for spiralin gene using *S*. *citri* infected fruit and leaf samples were done. The t-test was performed to compare the differences in measurement between inhibitors and no inhibitor control by ddPCR and qPCR assays. Statistical analyses were performed with IBM SPSS Statistics version 24.

### Ethics statement

The field manager of a private corporate farming company, owner/lessee, of the citrus orchard, gave permission to enter and sample the field several km east of Ducor in Tulare County, California. Moreover, in accordance with a Confidentiality Agreement between the U.S. Department of Agriculture, Agricultural Research Service and the owner/lessee of the property, the farming company approved publication of this report.

## Results

### qPCR assay

**Fig 1:**
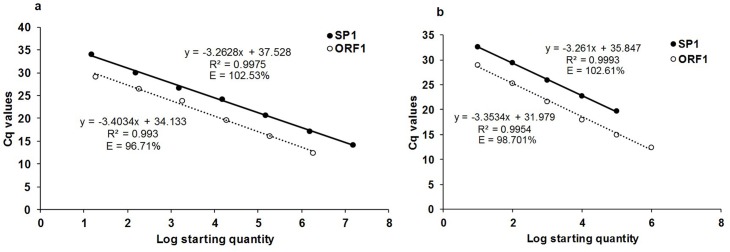
Calibration curve of qPCR assays with tenfold serially diluted SP1 and ORF1 Plasmid DNA (1.48E+07 to 1.48E+01 copies/μl and 1.80E+06 to 1.80E+01, respectively) and *Spiroplasma citri* DNA (10^−9^ to 10^−13^ for SP1 and 10^−14^ for ORF1) using SP1 (unbroken line) and ORF1 (broken line) primers. (a) The Plasmid DNA standard curve slope is -3.2628, equivalent to an efficiency of 102.53% for SP1 and -3.4034, equivalent to an efficiency of 96.71 for ORF1. (b) The *S*. *citri* DNA standard curve slope is -3.261, equivalent to an efficiency of 102.61% for SP1 and -3.3534, equivalent to an efficiency of 98.70% for ORF1.

The calibration curves showed the qPCR assays had good linearity with SP1 and ORF1 plasmid DNA (R^2^ = 0.997 and R^2^ = 0.993, respectively) over the dynamic range tested in both the plasmid DNA from 1.48E+07 to 1.48E+01 copies/μl and 1.80E+06 to 1.80E+01 copies/μl, respectively. The slopes were -3.2628 and -3.4034, equivalent to a PCR efficiency of 102.53% and 96.71% for SP1 and ORF1 respectively. The standard curve showed the sensitivities of the qPCR assays were 14.8 copies/20 μl and 18 copies/ 20 μl for SP1 and ORF1, respectively (Fig 1A, S2 Table). The *S*. *citri* DNA standard curve showed efficiencies of 102.61% (R^2^ = 0.9993, slope = -3.261) and 98.70% (R^2^ = 0.9954, slope = -3.3534) for SP1 and ORF1, respectively. The detection limits were 0.0001 ng and 0.00001 ng for SP1 and ORF1 respectively (Fig 1B, S3 Table). Based on above results and absolute quantification of plasmid DNA and *S*. *citri* DNA in ddPCR assays, the cut off values for *S*. *citri* positive samples were estimated to be 33 and 30 for SP1 and ORF1 primers, respectively.

### ddPCR assay and comparison with qPCR

**Fig 2:**
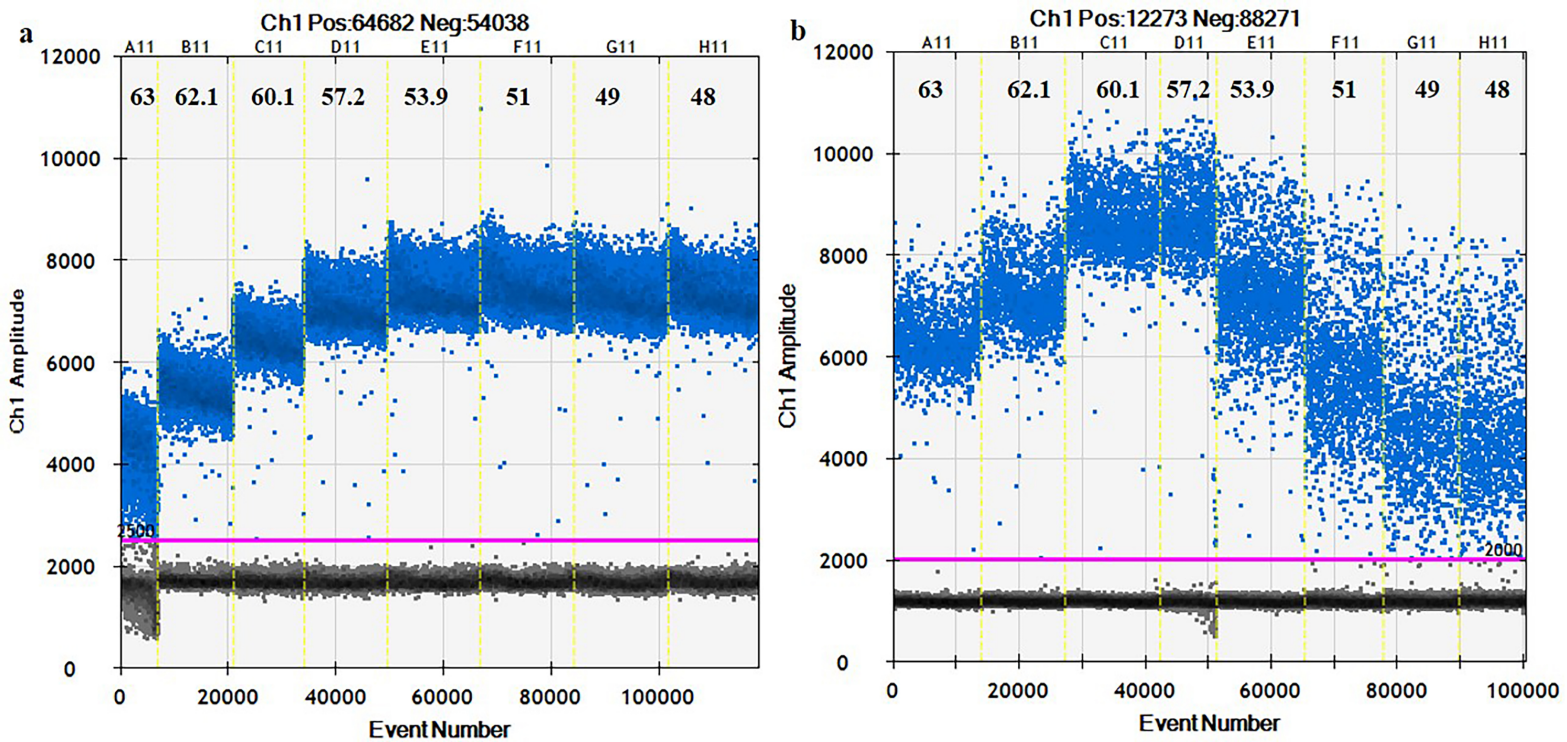
**Thermal gradient PCR for optimizing annealing temperature for (a) SP1 (b) ORF1 of *Spiroplasma citri***. Eight ddPCR reactions with an annealing temperature gradient ranged from 48°C to 63°C are divided by vertical dotted yellow lines. The pink line is the threshold, above which are positive droplets (blue) and below that are negative droplets (gray) without any target DNA.

The optimum annealing temperatures for ddPCR with SP1 and ORF1 primers were 54°C and 57°C, respectively (Fig 2A and 2B). These temperatures were selected on the basis of the greatest difference in fluorescence amplitude between *S*. *citri* DNA positives and negatives without nonspecific amplification.

**Fig 3:**
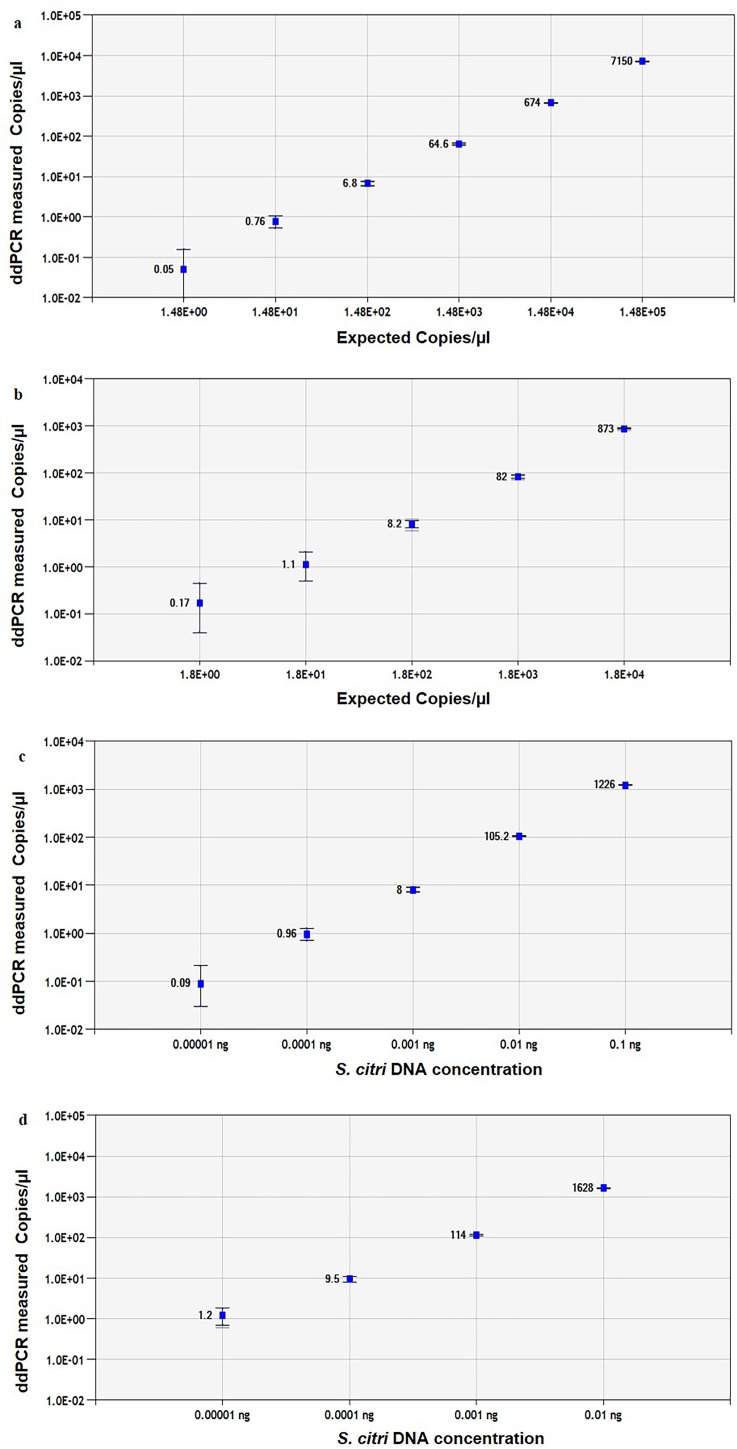
Linear regression of the ddPCR assays. The Pearson correlation coefficient are shown for the regression curves for *Spiroplasma citri* DNA from: (a) SP1 plasmid, y = 0.967x—182.68 (R^2^ = 1.0, *P* <0.0001); and (b) ORF1 plasmid y = 0.971x—26.109 (R^2^ = 1, *P* <0.0001); (c) SP1 *S*. *citri* DNA R^2^ = 0.9998, *P* <0.0001, and (d) ORF1 *S*. *citri* DNA R^2^ = 0.9992, *P* <0.0001. The inner error bars indicate the Poisson 95% confidence interval (CI) and the outer error bars show the total 95% CI of replicates.

The linear regression curve was made by plotting log10 transformed copy number concentrations measured by ddPCR against log10-transformed predicted values of serially diluted plasmid DNA (Fig 3). The ddPCR assay showed better linearity than qPCR for both SP1 and ORF1 Plasmid DNA, R^2^ = 1, *P* <0.0001 (Fig 3A); and R^2^ = 1, *P* <0.0001 (Fig 3B), respectively, between the target input amounts and measured values in the dynamic range of six and five orders of magnitude, respectively. Droplets were positively saturated at target concentrations >10^6^ copies/μl, making the Poisson algorithm invalid and resulting in a relative narrower dynamic range compared to qPCR. The sensitivity of ddPCR assay for SP1 and ORF1 plasmid was 1 copy/20μl reaction and 3.4 copies/20μl, respectively, which is more sensitive than qPCR (S2 Table).

The *S*. *citri* cell culture DNA showed good linearity in ddPCR assay than qPCR for both SP1 and ORF1, R^2^ = 0.9998, *P* <0.0001 (Fig 3C); and R^2^ = 0.9992, *P* <0.0001 (Fig 3D), respectively, over the dynamic range of five and four orders of magnitude, respectively. The sensitivity of ddPCR assay for SP1was 0.00001 ng, which was more sensitive than qPCR (0.0001 ng). The ORF1 primers had equal sensitivity for both qPCR and ddPCR assays (S3 Table).

### Diagnostic performance of ddPCR versus qPCR assays with field samples

Disparate results were obtained with qPCR Cq cutoff values of ≤ 33 and ≤ 30 for SP1 and ORF1 primers, respectively, between samples from leaf midrib versus fruit columella (Table 2). Leaf tissue samples met the positive threshold in only 4 of 50 trees with SP1 primers; whereas ORF1 met the threshold in 7 of the same 50-tree cohort. In contrast, SP1 identified 13 *S*. *citri* infected trees from the same 50-tree cohort using DNA extracted from fruit columella; while ORF1 detected the same number of *S*. *citri* infected trees. Results showed positive diagnosis of *S*. *citri* by ORF1 was more robust than SP1 and that detection of *S*. *citri* was greatly improved by testing fruit columella where pathogen titer was generally higher than in leaf tissue.

**Table 2:** Performance of qPCR and ddPCR assays using SP1 and ORF1 primers for detection of *Spiroplasma citri* in DNA extracted from fruit columella versus leaf tissue collected from 50 trees.

Sample analysis^a^	SP1	ORF1
	Positive/total tree (% Positive)	
**Leaf petiole midrib**		
qPCR	4/50 (8%)	7/50 (14%)
ddPCR	11/50 (22%)	14/50 (28%)
**Fruit columella**		
qPCR	13/50 (26%)	13/50 (26%)
ddPCR	13/50 (26%)	13/50 (26%)

^a^Samples harvested in November, 2016 from a 30-year old orchard of Spring Navel on Carrizo citrange rootstock in Tulare County, California.

ddPCR proved to be more accurate than qPCR to test for *S*. *citri* infection. Based on absolute quantitation of 1 copy of target in 20μl reaction, leaf midrib samples tested by SP1 detected 11 *S*. *citri* positive trees; whereas, ORF1 detected 14 infected trees from the same 50-tree cohort. Using DNA from fruit columella, 13 *S*. *citri* positive trees were found with SP1 primers; while the same 13 trees were found positive with ORF1 primers.

**Fig 4:**
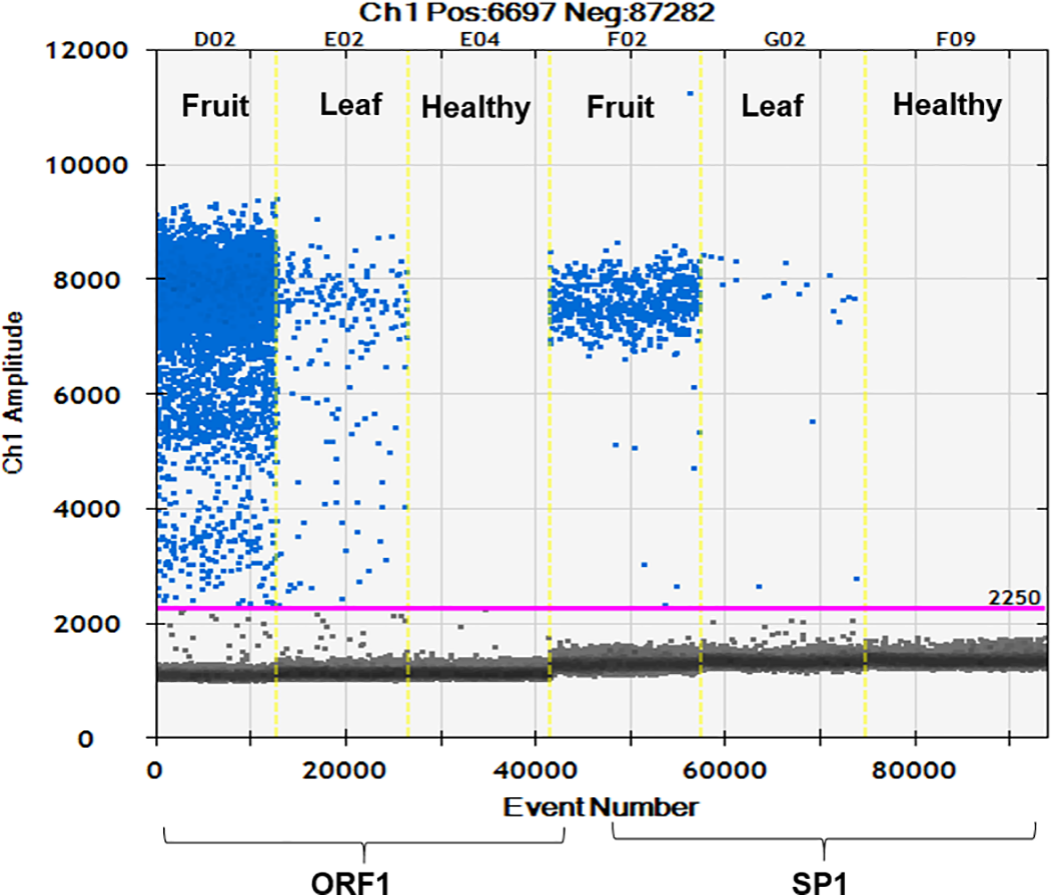
One dimensional plot of ddPCR assay showing *Spiroplasma citri* titer in fruit and leaf samples for ORF1 and SP1. The samples are divided by vertical dotted yellow lines. The unbroken pink line is the threshold, above which are positive droplets (blue) with PCR amplification and below which are negative droplets (gray) without any amplification.

Overall, *S*. *citri* infection was detected in 15 of 50 trees sampled (30%). qPCR and ddPCR both showed *S*. *citri* titer was higher in fruit columella than in leaf midrib tissue and that ORF1 primers yielded better positive readings for infection (Table 2). ORF1 primers resulted in identifying 14 of 15 *S*. *citri* infected trees for an overall accuracy of 98% from leaf midrib tissue samples. In contrast, fruit samples from the same trees resulted in identification of 13 *S*. *citri* infected trees with an accuracy of 96%. Since *S*. *citri* titer is higher in fruit columella than in leaf midrib tissue (Fig 4), this difference was attributed to sampling protocol where DNA extraction from three fruits versus 10 leaf petioles from the four quadrants of the tree canopy. Thus, as the area sampled increased, the chance of finding positive *S*. *citri* infected trees concomitantly increased.

**Fig 5:**
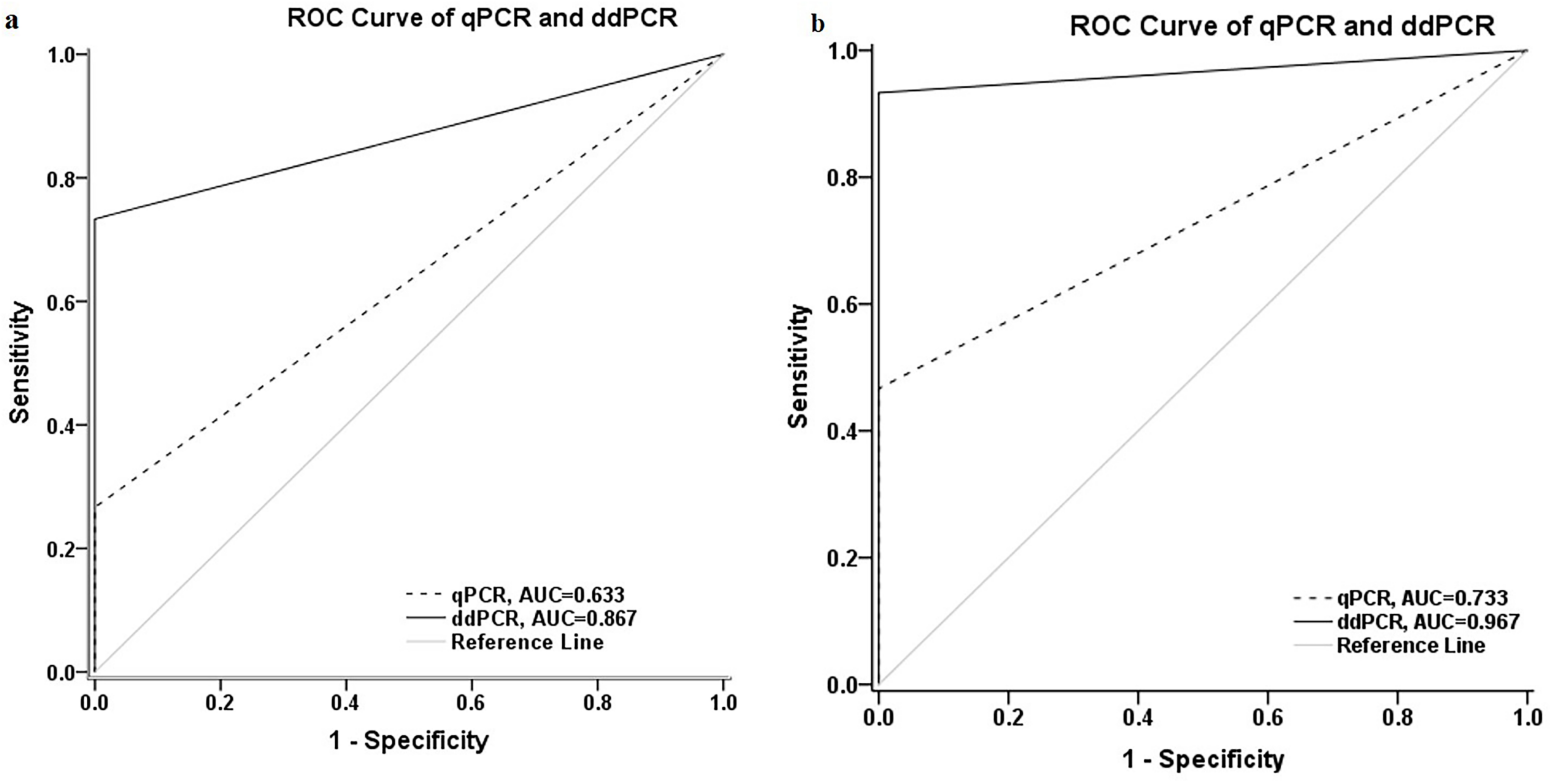
Diagnostic performance comparison between qPCR and ddPCR assays for citrus stubborn disease diagnosis. ROC curve indicates better diagnostic performance of ddPCR assays compared to the qPCR assays for differentiating between healthy and *S*. *citri* infected leaf samples with significantly (*P* <0.05) broader AUC 0.867 and 0.967 with (a) SP1 and (b) ORF1 primers, respectively.

**Fig 6:**
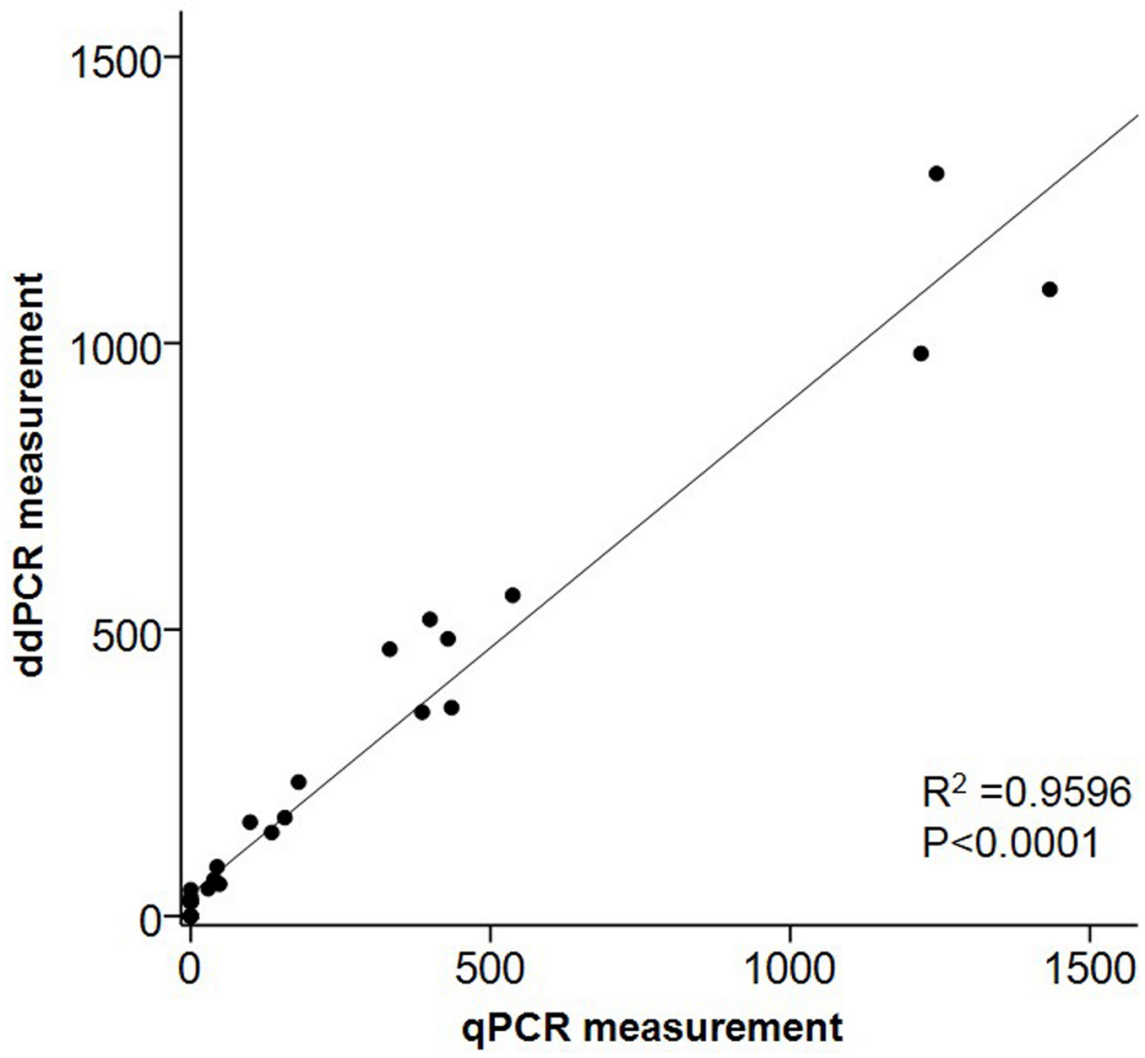
Correlation between ddPCR and qPCR measurements for spiralin gene using *S*. *citri* infected fruit and leaf samples. Both the assays were significantly (R^2^ = 0.9596, *P* <0.0001) correlated.

ROC analysis to compare diagnostic performance between ddPCR and qPCR assays of *S*. *citri* infected leaf samples showed that the area under curve (AUC) for ddPCR was 0.867 (standard error 0.071, 95% CI 0.728–1.0); whereas qPCR assay was 0.633 (standard error 0.094, 95% CI 0.450–0.817) (Fig 5A) for SP1 primers. For ORF1 primers, the area under curve (AUC) for ddPCR assay was 0.967 (standard error 0.038, 95% CI 0.892–1.0); whereas the qPCR assay was 0.733 (standard error 0.089, 95% CI 0.559–0.908) (Fig 5B). The AUC of the ddPCR assay was significantly (*P* <0.05) broader compared to the qPCR assay, indicating the ddPCR methodology was a more robust than qPCR for *S*. *citri* detection in field samples. The Pearson correlation and linear regression between qPCR and ddPCR assays for SP1 using *S*. *citri* infected fruit and leaf samples is strong (Fig 6).

### Influence of residual matrices on the qPCR and ddPCR assays

**Fig 7:**
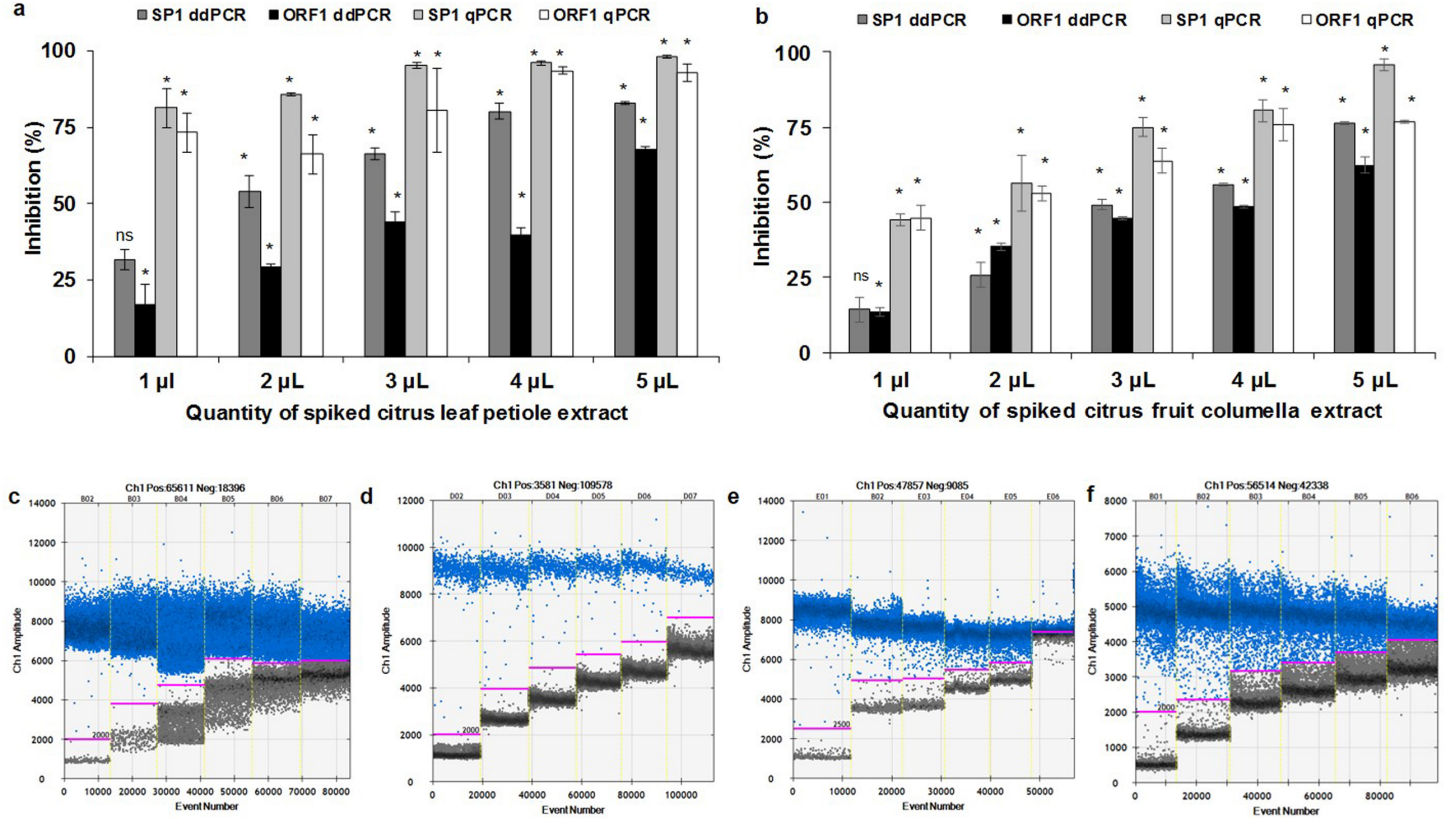
Influence of citrus leaf petiole and fruit columella extract on quantification of *S*. *citri* by ddPCR and qPCR assays for SP1 and ORF1 genes and 1-D plot of ddPCR reactions. Samples spiked with different quantity of (a) citrus leaf petiole, (b) fruit columella extracts and equal amount of *S*. *citri* plasmid DNA. Error bars denote standard error of inhibition between three replicates of each reaction. Asterisks (*) above each bar denote significantly (*P* <0.05) different measurements, compare to no inhibition control. The non-significant measurements denoted by ns. The 1-D plot show only one of three replication for SP1 and ORF1 with citrus leaf petiole (c & d respectively) and fruit columella extract (e & f respectively).

Both qPCR and ddPCR assays were inhibited with citrus leaf petiole and fruit columella extract but the ddPCR showed higher resilience to inhibition for the quantification of *S*. *citri* compare to qPCR (Fig 7A & 7B). The presence of inhibitors in qPCR assays resulted in a lower detection limit and an underestimation of *S*. *citri* titer in field samples which led to false negative results in the qPCR assays. In contrast, the key parameters affected by the presence of the residual matrices for the ddPCR were *S*. *citri* titer and fluorescent signal levels for both negative and positive droplets (Fig 7C, 7D, 7E & 7F). Fluorescent signals of both positive and negative droplets were increased with increasing amounts of spiked citrus leaf petiole and fruit columella extracts.

### Reproducibility and repeatability of the ddPCR assays for field samples

The intra-assay coefficient of variation for fruit and leaf samples was 1.1% and 8.8%, respectively, using ORF1 primers; and 2.7% and 14.7%, respectively, using SP1 primers. The inter-assay coefficient of variation for fruit and leaf samples was 2.1% and 5.1%, respectively, using ORF1 primers; and 1.1% and 21.8%, respectively, using SP1 primers. The higher titer of *S*. *citri* in fruit versus leaf samples resulted in lower CV values from fruit samples (Table 3).

**Table 3:** Repeatability (Intra-assay variation) and reproducibility (Inter-assay variation) of ddPCR method for detection of *Spiroplasma citri*.

**Assay**	**Gene**	**Sample**	**ddPCR** ^a^			
**Inter-assay variability**			**Assay 1**^**b**^	**Assay 2**^**b**^	**Assay 3**^**b**^	**CV%**^c^
	SP1	Fruit	55	55	53.9	1.1
		Leaf	1.6	1.1	1.6	21.8
	ORF1	Fruit	761.3	750.7	730.3	2.1
		Leaf	15.2	16.8	15.6	5.1
**Intra-assay variability**			**Replicate 1**	**Replicate 2**	**Replicate 3**	
	SP1	Fruit	54.2	54	56.7	2.7
		Leaf	1.7	1.7	1.3	14.7
	ORF1	Fruit	735	721	735	1.1
		Leaf	15.3	17.1	14.4	8.8

^a^ Values reflect copies/μl ddPCR reaction

^b^ Average of three replicates

^c^ CV means coefficient of variation

## Discussion

Early detection of citrus pathogens is crucial to initiate management efforts to limit or prevent further spread of the disease. In the present study, the assessment of ddPCR in comparison to qPCR for the early and quantitative detection of single and multi-copy gene targets of *S*. *citri* in fruit and leaf samples were carried out. The single copy gene SP1, may not be reliably used for the detection of *S*. *citri* due to low titer and erratic distribution of the pathogen. Since ORF1 is a multi-copy gene, it significantly improved the sensitivity of *S*. *citri* detection in culture and field samples. The utility of a direct (ddPCR) rather than a relative (qPCR) DNA-based measurement to quantify *S*. *citri* or other pathogens is very important, particularly if proven precise and reliable. A major limitation of qPCR is the quantitative data generated are only as accurate as the standards used. The external calibration curve for absolute quantification is usually obtained from a series of dilutions of known concentrations, which is often an expensive, laborious, and time-consuming process if it is not readily available [20]. Several studies relative to food, clinical, and environmental samples showed that ddPCR is less susceptible to PCR inhibitors [21, 22, 23, 24]. The ddPCR has several advantages over qPCR such as absolute quantification without the need of calibration curve, improved accuracy, less prone to inhibitors, reliability and reproducibility between inter and intra assays.

In this study, linearity, dynamic range, sensitivity, tolerance to residual matrix inhibitors and diagnostic performance of ddPCR and qPCR was compared for quantitative detection of *S*. *citri*. Both ddPCR and qPCR showed a high degree of linearity with plasmid and *S*. *citri* DNA. The key limitations of ddPCR in comparison to qPCR is the upper quantification limit for the ddPCR was lower and higher concentration of template resulted in saturation of positive droplets [17]. This led to significant loss of linearity at high concentration [25]. Despite this limitation, absolute quantification of the *S*. *citri* showed good correlation with real-time PCR. The large scale partitioning involved in ddPCR allows a greater precision and sensitivity in comparison with real-time PCR [25]. The positive counts together with the Poisson’s distribution was used to determine a high confidence measurement of the targeted molecules [26, 27, 28]. In addition, partitioning reactions in picoliter droplets allows ddPCR to have lesser interference with PCR inhibitors.

The qPCR positive cutoff Ct values of 33 and 30 for SP1 and ORF1, respectively, were derived empirically from target transcript and cell culture experiments and, as such, were unambiguous and the data presented showed that the qPCR procedure for *S*. *citri* detection was robust. However, data derived from field samples can still result in ambiguous results when Ct values are at the upper limits. False positive and negatives are always a critical concern and must be accounted for due to variables such as pathogen titer, erratic distribution, sampling error, PCR inhibitors, poor sample collection, seasonal growth variations, climate, horticultural conditions, etc. For these reasons, it is encouraged that several gene targets for detection be used and that an alternative method such as ddPCR be available if needed. Furthermore, although sampling leaf petioles for *S*. *citri* is more convenient than fruit columella where *S*. *citri* titer is higher, it is advisable to take samples from fruit if present during periods of low pathogen titer. PCR inhibition was observed in the citrus leaf extract causing some qPCR false negatives with citrus leaf samples; whereas ddPCR was more tolerant to PCR inhibition. The copy number determined by ddPCR is fewer than calculated by the empirical formula for the cloned plasmids. Measurements of DNA by optical density lack accuracy in distinguishing intact and fragmented nucleic acid particles. ROC analysis showed that AUC was greater for ddPCR than qPCR for the samples categorized into diseased and non-diseased status. The higher AUC indicates better sensitivity and better diagnostic performance with regard to false positive or negative results for *S*. *citri* diagnosis compared to qPCR. The ddPCR assay exhibited repeatable and reproducible quantitative results without the need of an external curve, especially at low target concentration for *S*. *citri* positive leaf samples collected from the field. The high level of disease diagnosis by both methods was remarkable despite sampling during the winter when foliar growth stops and pathogen development slows or stops due to low temperature. The qPCR is cheaper and provides better throughput, however, ddPCR is a useful technique for calibrating qPCR standards to produce much more accurate standard curves. Individual samples showing high inhibition can be successfully assayed by ddPCR. This combination offers more robust, accurate, high throughput, affordable, and sensitive quantitation of plant pathogens.

## Conclusions

This work is the first to provide a range of information such as linearity, dynamic range, repeatability, reproducibility, tolerance to residual matrix inhibitors and diagnostic performance on the utility of ddPCR for absolute quantification of *S*. *citri*. Our results demonstrated the potential of the ddPCR assay compared to established qPCR assay. This technology is more sensitive for detection of low quantities of target DNA than qPCR with greater tolerance to PCR inhibitors. A very low copy number of *S*. *citri* target can be successfully detected in ddPCR using the housekeeping gene that helps in reliable early detection of pathogen. The ddPCR targeting a multi-copy gene such as SpV1 ORF1, significantly improved the *S*. *citri* detection in culture and field samples. The increased sensitivity can have additional value for early detection of the pathogen. The ddPCR is a more robust method for detection of *S*. *citri* in asymptomatic citrus tissues in a slow or inactive period of infection. Finally, when *S*. *citri* titer is at its lowest seasonal levels, it is best to use SpV1 ORF1 rather than spiralin as the target and ddPCR rather than qPCR, respectively, for CSD diagnosis.

## Supporting information

S1 Table Primers used for *Spiroplasma citri* SP1 and ORF1 gene cloning for standard curve. (PDF)

S2 Table Quantitative data of *Spiroplasma citri* plasmid DNA with SP1 and ORF1 primers in qPCR and ddPCR assays. (PDF)

S3 Table Quantitative data of *Spiroplasma citri* cell culture DNA with SP1 and ORF1 primers in qPCR and ddPCR assays. (PDF)
