# Supplementary material for: Application of droplet digital PCR for quantitative detection of Spiroplasma citri in comparison with real time PCR
Source: PLoS One. 2017 Sep 14;12(9):e0184751. doi: 10.1371/journal.pone.0184751 (PMC5599046; doi:10.1371/journal.pone.0184751)
Supplement: S1 Table — (PDF) [file pone.0184751.s001.pdf]

S1 Table. Primers used for *Spiroplasma citri* SP1 and ORF1 gene cloning for standard curve

Target Gene	Primer	Sequence (5'-3')	Amplicon length (bp)	Reference
Spiralin	Spiralin F	GTCGGAACAACATCAGTGGT	674	Yokomi et al., 2008
	Spiralin R	TGCTTTTGGTGGTGCTAATG		Yokomi et al., 2008
Prophage	Prophage F	TGCGATATTTGGAACTGGTTT	533	this study
	Prophage R	TCATAAACAACTCCGGTTGAAT		this study
