# Supplementary material for: Application of droplet digital PCR for quantitative detection of Spiroplasma citri in comparison with real time PCR
Source: PLoS One. 2017 Sep 14;12(9):e0184751. doi: 10.1371/journal.pone.0184751 (PMC5599046; doi:10.1371/journal.pone.0184751)
Supplement: S2 Table — (PDF) [file pone.0184751.s002.pdf]

S2 Table. Quantitative data of *Spiroplasma citri* plasmid DNA with SP1 and ORF1 primers in qPCR and ddPCR assays

SP1 Plasmid							ORF1 Plasmid						
Calculated copies/µl[a]	qPCR			ddPCR			Calculated copies/µl[a]	qPCR			ddPCR		
	Mean Cq	SD[b]	Log SQ[c]	Mean[d]	Poisson SEM[e]	Total SEM		Mean Cq	SD[b]	Log SQ[d]	Mean[d]	Poisson SEM[e]	Total SEM
1.48E+07	14.21	0.014	7.170	NA	NA	NA	1.80E+06	12.49	0.119	6.255	NA	NA	NA
1.48E+06	17.23	0.053	6.170	NA	NA	NA	1.80E+05	16.17	0.104	5.255	NA	NA	NA
1.48E+05	20.73	0.096	5.170	143000	124.23	147.46	1.80E+04	19.69	0.167	4.255	17460	9.53	40.43
1.48E+04	24.36	0.274	4.170	13480	5.49	7.51	1.80E+03	23.97	0.092	3.255	1640	3.18	6.06
1.48E+03	26.78	0.693	3.170	1292	1.41	3.18	1.80E+02	26.60	0.642	2.255	164	0.78	1.36
1.48E+02	30.03	0.158	2.170	136	0.43	0.58	1.80E+01	29.20	0.223	1.255	22	0.47	0.47
1.48E+01	34.12	0.658	1.170	15.2	0.15	0.15	1.80E+00	NA	NA	NA	3.4	0.12	0.12
1.48E+00	NA[g]	NA	NA	1	0.04	0.04	NTC	0	0	0	0	0	0
NTC[f]	0	0	0	0	0	0							

[a] Values reflect copies/µl of calculated serial dilutions of positive plasmid DNA standard.

[b] SD means standard deviation.

[c] SQ means starting quantity. Data represent the mean of each dilution tested in triplicate.

[d] Values reflect copies/20 µl ddPCR reaction. Data represents the ddPCR values from merged triplicates of each dilutions.

[e] SEM means standard error of mean.

[f] NTC means no template control.

[g] NA means not applicable.
