# Supplementary material for: Application of droplet digital PCR for quantitative detection of Spiroplasma citri in comparison with real time PCR
Source: PLoS One. 2017 Sep 14;12(9):e0184751. doi: 10.1371/journal.pone.0184751 (PMC5599046; doi:10.1371/journal.pone.0184751)
Supplement: S3 Table — (PDF) [file pone.0184751.s003.pdf]

S3 Table. Quantitative data of *Spiroplasma citri* cell culture DNA with SP1 and ORF1 primers in qPCR and ddPCR assays

S.citri DNA SP1						*S.citri* DNA ORF1				
DNA concentration[a]	qPCR		ddPCR			qPCR		ddPCR		
	Mean Cq	SD[b]	Mean[c]	Poisson SEM[d]	Total SEM	Mean Cq	SD	Mean	Poisson SEM	Total SEM
1ng	19.7	0.087	NA[e]	NA	NA	12.44	0.166	NA	NA	NA
0.1ng	22.69	0.107	24520	8.37	8.37	15.03	0.087	NA	NA	NA
0.01ng	25.89	0.11	2104	1.73	2.31	17.97	0.226	32560	17.32	17.32
0.001ng	29.38	0.182	160	0.49	0.73	21.7	0.186	2280	33.65	33.65
0.0001ng	32.66	0.031	19.2	0.16	0.16	25.31	0.231	190	0.87	0.87
0.00001ng	0	0	1.8	0.05	0.05	29	0.346	24	0.32	0.35
Healthy	0	0	0	0	0	0	0	0	0	0

[a] Values reflect concentration of *S. citri* DNA used for serial dilution.

[b] SD means standard deviation.

[c] Values reflect copies/20 µl ddPCR reaction. Data represents the ddPCR values from merged triplicates of each dilutions.

[d] SEM means standard error of mean.

[e] NA means not applicable.
